# Origins of pressure dependent permeability in unconventional hydrocarbon reservoirs

**DOI:** 10.1038/s41598-023-33601-5

**Published:** 2023-05-02

**Authors:** John J. Valenza, Pavel Kortunov, Shehab Alzobaidi, William Horn, Brian Crawford

**Affiliations:** 1 Research Division, ExxonMobil Technology and Engineering Co., Annandale, NJ 08801 USA; 2 ExxonMobil, Upstream Research Company, Spring, TX 77389 USA; 3 Formerly of ExxonMobil, Upstream Research Company, Spring, TX 77389 USA

**Keywords:** Geophysics, Mineralogy, Soft materials, Condensed-matter physics, Structure of solids and liquids

## Abstract

Unconventional hydrocarbon assets represent a rapidly expanding proportion of North American oil and gas production. Similar to the incipient phase of conventional oil production at the turn of the twentieth century, there are ample opportunities to improve production efficiency. In this work we demonstrate that pressure dependent permeability degradation exhibited by unconventional reservoir materials is due to the mechanical response of a few commonly encountered microstructural constituents. In particular, the mechanical response of unconventional reservoir materials may be conceptualized as the superposed deformation of matrix (or ~ cylindrical/spherical), and compliant (or slit) pores. The former are representative of pores in a granular medium or a cemented sandstone, while the latter represent pores in an aligned clay compact or a microcrack. As a result of this simplicity, we demonstrate that permeability degradation is accounted for through a weighted superposition of conventional permeability models for these pore architectures. This approach permits us to conclude that the most severe pressure dependence is due to imperceptible bedding parallel delamination cracks in the oil bearing argillaceous (clay-rich) mudstones. Finally, we demonstrate that these delaminations tend to populate layers that are enriched with organic carbon. These findings are a basis for improving recovery factors through the development of new completion techniques to exploit, then mitigate pressure dependent permeability in practice.

## Introduction

Under the dual constraint of minimizing the cost, and associated carbon footprint of hydrocarbon production, unconventional oil and gas reserves represent an attractive class of energy assets^1,2^. Given the nascent state of unconventional oil production, there is an opportunity to reduce the associated carbon footprint^3,4^, by improving meager recovery factors^5^. Unconventional oil production largely consists of shale oil. As a result, this study focuses on rocks from shale oil assets in the Bakken, Uinta Piceance, and Permian basins. In general, shale, also commonly referred to as mudstones, exhibits much lower permeability than more conventional reservoir materials, like sandstone. This is the case because mudstones contain significantly more fine sediment like silt and clay. Given the low permeability of these so-called *tight rocks* oil production is facilitated by extended reach horizontal drilling, and intensive hydraulic fracturing. Under these circumstances low permeability rock (*k* < 10^–18^ m^2^) plays a dominant role in dictating both production rates and cumulative production^6,7^.

Previous studies focused on establishing a relationship between microstructural characteristics, and the ultra-low *k* exhibited by tight rocks^8–10^. In addition, numerous studies^11–16^ show that unconventional reservoir materials exhibit a broad spectrum of permeability degradation, or pressure dependent permeability (PDP), as the net confining stress increases. More specifically, the permeability of unconventional reservoir materials is reduced by a factor in the range of 2–100 over a range of confining stress relevant to hydrocarbon production. In contrast, conventional sandstones exhibit at most a 10% permeability reduction under the same conditions. This behavior significantly delays, and in many cases limits total fluid production^14,17^.

In this work we describe the microstructural features, and associated mechanisms that underlie pressure dependent porosity (PDPo) and PDP in unconventional reservoir materials. In our experiments the governing pressure is the net confining pressure, *p*_CS_ = *p*_C_ − *p*_P_, which is the difference between the hydrostatic confining pressure (e.g., mimicking the overburden), *p*_C_, and the pressure in the pore fluid, *p*_P_. In practice, the *p*_CS_ increases when fluid is extracted from rock by lowering *p*_P_ at a free surface (e.g., at the interface with a pipe or in a fracture). The approach that follows consists of employing effective medium approximations to account for the equilibrium poromechanical response of a representative spectrum of tight rocks by assuming the behavior is due to the deformation of grain supported (or ~ spherical) pores, compliant (or slit) pores, or mixtures thereof^18^. Conventional microstructural analogues for the former are a granular medium or a cemented sandstone, while that for the latter is a highly aligned clay compact or a microcrack. Since the primary sediments in tight rocks are sand or carbonate sediment, and clay/silt with associated organic matter (e.g., grain supported, or clay, respectively) we use the mineralogy as a proxy for the proportion of each pore type. In turn, this work is focused on assessing the ability to account for PDP with conventional permeability models for the two types of pores*.* Finally, the effective medium theory is constrained by state-of-the-art electron probe microanalysis, and nanoindentation measurements to determine the composition, and mechanical properties associated with the main microstructural constituents, respectively.

## Materials and methods

This work was motivated in part by the experimental data presented in refs.^12^ and 14, and the analysis presented in ref.^18^. The data sets from these references are valuable because they measure pressure dependent porosity (PDPo) *and* permeability (PDP). A secondary objective of this work is to demonstrate the utility of this complimentary information for highlighting the contribution of common microstructural features to the observed pressure dependence. In addition to the data presented in both of those references, we utilize new results from these techniques. More specifically, the mechanical testing protocol described in ref.^14^ was utilized to collect data on Bakken and Mancos shale samples, whereas the nuclear magnetic resonance technique^12^ was utilized to collect data from various Wolfcamp samples across the Permian basin*.*

Both experimental protocols utilize horizontal cores, where the bedding is parallel to the cylinder axis, and all testing was performed under conventional hydrostatic confining pressure, *p*_C_. The cylindrical core with a porous frit, and a fluid distribution platen on either end is isolated from the confining pressure with an impermeable jacket (see Supplementary Information, Fig. S1). This arrangement permits independent control of *p*_P_, and *p*_C_. In all cases fluid flow is along the cylinder axis, and thus parallel to the bedding planes. A detailed description of the experimental protocol is presented in refs.^12^ and 14, but we provide a brief synopsis below.

### Pressure dependent porosity: PDPo

Proton (H^+^) NMR provides a measure of $\phi_0$, or pressure dependent porosity by saturating the sample with toluene, or oil, then associating the change in proton count to fluid filled porosity. In the case of the mechanical testing protocol, standard pycnometry is utilized to infer the initial porosity from the ratio of the bulk, *ρ*_B_, and grain density, *ρ*_S_, of the rock^19^ $\phi_0$ = 1 − *ρ*_*B*_/*ρ*_S_. In this case the pressure dependent porosity is assumed to be equivalent to the change in sample volume, which is determined from the axial and circumferential strain (see Supplementary Information, Fig. S1).

### Pressure dependent permeability: PDP

Both experimental protocols are based on the conventional steady state permeability measurement technique. This technique consists of pressing a fluid through the sample at a constant flow rate, *Q*, then monitoring the pressure drop, Δ*p*_P_, across the sample to identify a steady state. The pore fluid varies between the protocols with nitrogen gas used in the mechanical testing protocol, and toluene/degassed oil utilized in the NMR protocol. When gas is used it is necessary to account for the compressibility of the gas, as discussed in ref.^14^. Thus once a steady state is achieved the permeability, *k*, is given by^20^:

1 $$k_{i} = \frac{{2Q\eta LX_{i} }}{{\Delta p_{P} }}$$

where $\eta$ is the fluid viscosity, *L* is the length of the core, Δ*p*_P_ = *p*_P,I_- *p*_P,O_, *X*_l_ = 1 when a incompressible fluid is used or $X_{g} = p_{P,I} /\left( {p_{P,I} + p_{P,O} } \right)$ when a gas is used, and the subscripts *I* and *O* correspond to the pore pressure at the sample inlet or outlet, respectively.

### Electron probe micro-analysis (EMPA)

The measurement consists of rastering a 15 keV electron beam over a polished surface (see Supplemental Information—Notes for sample preparation) while detecting the secondary X-rays emitted with two elemental dispersive spectroscopy (EDS) detectors. The averaged X-ray spectra are converted to elemental mass fraction with a matrix corrected ZAF approach^21^, using mineral standards: Al_2_O_3_, CaCO_3_, NaCl, FeS_2_, K-feldspar, MgO, SiO_2_, FeP, and TiO_2_. Assuming the earth’s crust is predominantly composed of these minerals we determine the elemental composition consisting of Al, Ca, C, Cl Fe, K, Mg, Na, O, S, Si, and Ti. Of these we use Al, C, Ca, Fe, Si, as proxies for the major phases found in unconventional reservoir materials, those being, clay, organic or inorganic carbon, pyrite, and silt/sand, respectively. It should be noted that none of the samples utilized in this work, contained a considerable amount (> 5% mass fraction) of any of the elements not contained in this limited list. The elemental maps for the indicated elements are created with ImageJ using the merge channels function. This function converts the 16-bit intensity maps for the individual elements to the appropriate intensity of the indicated color (Red, Blue, Green, Yellow, Cyan, and Magenta). The scaled color maps are combined, then saved as a 24-bit RGB image.

### Nanoindentation

The nanoindentation measurements are performed with a Bruker Hysitron TI premier and pyramidal Berkovitch diamond indenter with a tip radius on the order of 10 nm. The measurements consist of bringing the tip into contact with the sample, then indenting the sample while simultaneously measuring the load and tip displacement. The roughly hemispherical volume interrogated by the indentation is characterized by a radius that is 4–6 times the indentation depth^22^. Our experiments consist of indentation depths from 1 to 4 µm, so the measurements have a spatial resolution ~ 10 µm. This length scale is nominally an order of magnitude larger than the grains that make up the sample (e.g., silt and clay), and at least two orders of magnitude smaller than most microstructural features of interest. Where these conditions are met, we quantify the compliance of the microstructural constituents of interest in order to inform the effective medium theory. We use the elemental maps as a guide to identify areas that are representative of the microstructural constituents, then perform the nanoindentation on the same polished, carbon-coated, samples that are prepared for EPMA (see Supplemental Information—Methods).

Numerous references discuss the process for inferring local mechanical properties from the load versus displacement observed during an indentation measurement. We utilize the commonly employed Oliver and Pharr technique^23^, one aspect of which is relating the indentation depth to the contact area, *A*_C_, between the indenter and the sample. The indentation modulus, $M = S\sqrt \pi /2\sqrt {A_{C} }$, is a proxy for the bulk modulus, $K = M\left( {1 - \nu^{2} } \right)/3\left( {1 - 2\nu } \right)$, where, *S* is the slope of the unloading curve and *ν* is poisson’s ratio. *S* is determined from the linear portion of the unloading curve observed as unloading begins^23^. For the values of *ν* (0.2–0.35) commonly exhibited by these rocks, and the mineral components *K* ≈ *M*/1.5.

### Nitrogen gas adsorption/desorption

Nitrogen gas sorption was performed on roughly 1 g of pulverized sample held in a sample tube of known volume. The sample pretreatment consists of degassing at 80 °C. The measurement consists of pulling a high vacuum on the sample, imposing an isotherm by submerging in liquid nitrogen (− 196.15 °C), then monitoring the pressure evolution as the sample tube is dosed with known amounts of N_2_ gas. The difference between the measured pressure increase and that expected from the equation of state indicates the fraction of gas that is either physisorbed or condensed in the sample porosity; where physisorption is dominant at low pressure and capillary condensation is dominant at high pressure^24^. We use the kelvin equation to determine the pore size distribution from the volume of gas that evaporates over a given infinitesimal reduction in gas pressure (e.g., below the saturation vapor pressure). The kelvin equation relates the curvature of the liquid/vapor meniscus to the gas pressure, and we assume the meniscus curvature is commensurate with the associated pore size. Analysis of the desorption branch provides insight on the porosity accessible through pores of a given size. As a result, the peak in the derivative of the cumulative pore size distribution (e.g., sequentially reducing the gas pressure through the capillary regime) provides an estimate of the characteristic pore size that controls transport (see supplementary information, Fig. S3).

### Mercury intrusion capillary porosimetry

Mercury intrusion was performed with a micromeritics Autopore IV 9500. Prior to the measurement, the rock was granulated and sieved to yield particles with a diameter in the range 0.1–0.3 cm. The mercury intrusion volume was logged at 205 pressures in the range 0.0003–414 MPa. The Washburn equation, with known values of the contact angle and surface tension of mercury/air^19^, was used to relate pressure to the pore volume accessible through pores that range in size from 3.60 to 0.003 µm, respectively. As with the nitrogen gas sorption measurements, a rough estimate of the characteristic pore size is determined from the peak in the derivative of the cumulative intrusion volume (see supplementary information, Fig. S3).

## Theory and analysis

### Poromechanical response in the low frequency regime

The experiments are performed in the quasi-static (e.g., low frequency) regime. This justifies the assumption that the porous body responds as if it were drained^25^, and thus *p*_P_ evolves to a constant gradient between that imposed on either face during the permeability measurement (e.g., *p*_I_ and *p*_O_). The primary objective of this work is to account for the contribution of each pore type to the mechanical response, and by extension the permeability of the rock. A concise description of our approach is outlined below, largely focused on the primary analytical result utilized to interpret the experimental data, while a more complete description of the analysis is presented in the notes section of the supplementary information.

In this approach, the porosity is divided into stiff, or matrix supported, *ϕ*_Mat_, and compliant or slit pores, *ϕ*_Slit,_ $\phi = \phi_{Mat} + \phi_{Slit}$. The analysis outlined in the supplementary information demonstrates that the change in the matrix porosity, Δ*ϕ*_Mat_, is due to the linear elastic response of the solid skeleton, and that of the porous medium. In contrast, Δ*ϕ*_Slit_ is modulated by the change in the compliance associated with increasing the contact area as the slit aperture is reduced; This guarantees that the response is nonlinear^26^ (see supplementary information—Notes). The total change in porosity is:

2 $$\Delta \phi = \phi \left( {p_{CS} } \right) - \phi_{0} = \left( {C_{S} - C_{P\infty } } \right)p_{CS} - \phi_{Slit,0} \left[ {1 - \exp \left( { - \frac{{\partial C_{P} }}{{\partial \phi_{Slit} }}p_{CS} } \right)} \right]$$

where *C* = 1/*K*, indicates a compliance, and the subscripts, *S* and *P*, indicate properties of the solid phase, and the drained porous body. The distinction between the two compliances is that the skeleton of the porous medium is made up by the solid phase. Therefore, *C*_*S*_ < *C*_*P*_ (or *K*_*S*_ > *K*_*P*_), where *C*_P∞_ is the high confining pressure limit of *C*_P_^18^. The first term on the right hand side of Eq. (2) indicates that as the pressure increases there is a reduction in porosity associated with the difference between the compaction of the porous body, and the solid phase. The remaining terms account for porosity reductions associated with the closure of slit like pores. The former contribution exhibits a linear, while the latter an exponential dependence on net confining pressure. Equation (2) also demonstrates the utility of simultaneously measuring pressure dependent porosity and permeability, and complimenting these measurements with local measures of *C*_*P*_ and *C*_*S*_. Accordingly, we assess the viability of constraining the first term in Eq. (2) utilizing effective medium theory informed by nanoindentation. This would permit an accurate inference of $\phi_{Slit}$ and the non-linear parameter ∂*C*_*P*_/∂$\phi_{Slit}$ from the pressure dependent porosity. To estimate *C*_P∞_ we use the average value of the Hashin-Strikhman bounds^27^

3 $$K_{L,U} = \left\langle K \right\rangle - \frac{{\phi_{1} \phi_{2} \left( {K_{2} - K_{1} } \right)^{2} }}{{\left\langle {\overline{\tilde{K}} } \right\rangle + \frac{4}{3}G_{1,2} }}$$

where *ϕ﻿* is the phase fraction, *G* is the shear modulus, the subscript 2 (1) corresponds to the material with the upper (U) (lower(L)) bulk modulus, and the values in the angle brackets indicate a weighted average, $\left\langle K \right\rangle = \phi_{1} K_{1} + \phi_{2} K_{2}$, or $\left\langle {\overline{\tilde{K}}} \right\rangle = \phi_{1} K_{2} + \phi_{2} K_{1}$.

#### Permeability models

We assume the permeability of the sample is a linear superposition of that attributed to the two pore types, $k(p) = k_{Mat} (p) + k_{Slit} (p)$. Since both models result in a formula that takes the form:

4 $$k_{i} (p_{CS} ) = \phi_{{\text{i}}} (p_{CS} )B_{i}$$

the corresponding pressure dependent permeability may be determined by accounting for the pressure dependent porosity. The proportionality between *k*_i_ and *ϕ*_i_ in Eq. (4) for matrix supported pores, *B*_Mat_ = r_P_^2^/*χ*, yields the Carmen-Kozeny equation^28^. Here *r*_P_ is the characteristic pore size, and the tortuosity, is set to *χ* = 5. This approach assumes *r*_P_ is constant, and thus predominantly dictated by the characteristic grain size. Therefore, the pressure dependent permeability associated with deformation of *ϕ*_*Mat*_ is simply a linear function of porosity (Eq. (4)) with slope *r*_*p*_^2^/χ. Since, *ϕ*_*Mat*_ scales linearly with pressure, *k*_*Mat*_ also exhibits a linear dependence on pressure, like that exhibited by a conventional sandstone^29^. The permeability of slit like pores with aperture, *b*, is approximated by the appropriately named slit model, *B*_Slit_ = *b*^2^/12^30^. Therefore, *k*_Slit_ also exhibits a linear dependence on porosity. Since the porosity attributed to slit pores exhibits an exponential dependence on pressure (Eq. (2)), the permeability also exhibits the same behavior. Nonetheless, the similarities between the permeability models (Eq. (4)) for grain supported or slit pores demonstrates that a detailed understanding of the contribution of each pore type to changes in the permeability can only be achieved when the poromechanical response of the sample is characterized (e.g. using Eq. (2)).

In the supplementary information we show that the relatively high compliance of the slit pores, especially at low *p*_CS_, is due to variation in *b* which is the origin of the variation in *ϕ*_*Slit*_

5 $$\phi_{Slit} \approx \frac{NbD}{{A_{S} }} \approx \frac{4Nb}{{\pi D}}$$

where *N* is the total number of slit pores and *A*_S_ = *πD*^2^/4 is the cross-sectional area of the sample. In theory both *N* and *A*_S_ may be pressure dependent. However, substituting Eqs. (5) into (4) shows that the permeability exhibits a much stronger dependence on the slit aperture, *b*, so we assume *N* and *A*_S_ are constant.

6 $$k_{Slit} \approx \frac{{Nb^{3} }}{3\pi D}$$

## Results and discussion

### EPMA

**Figure 1:**
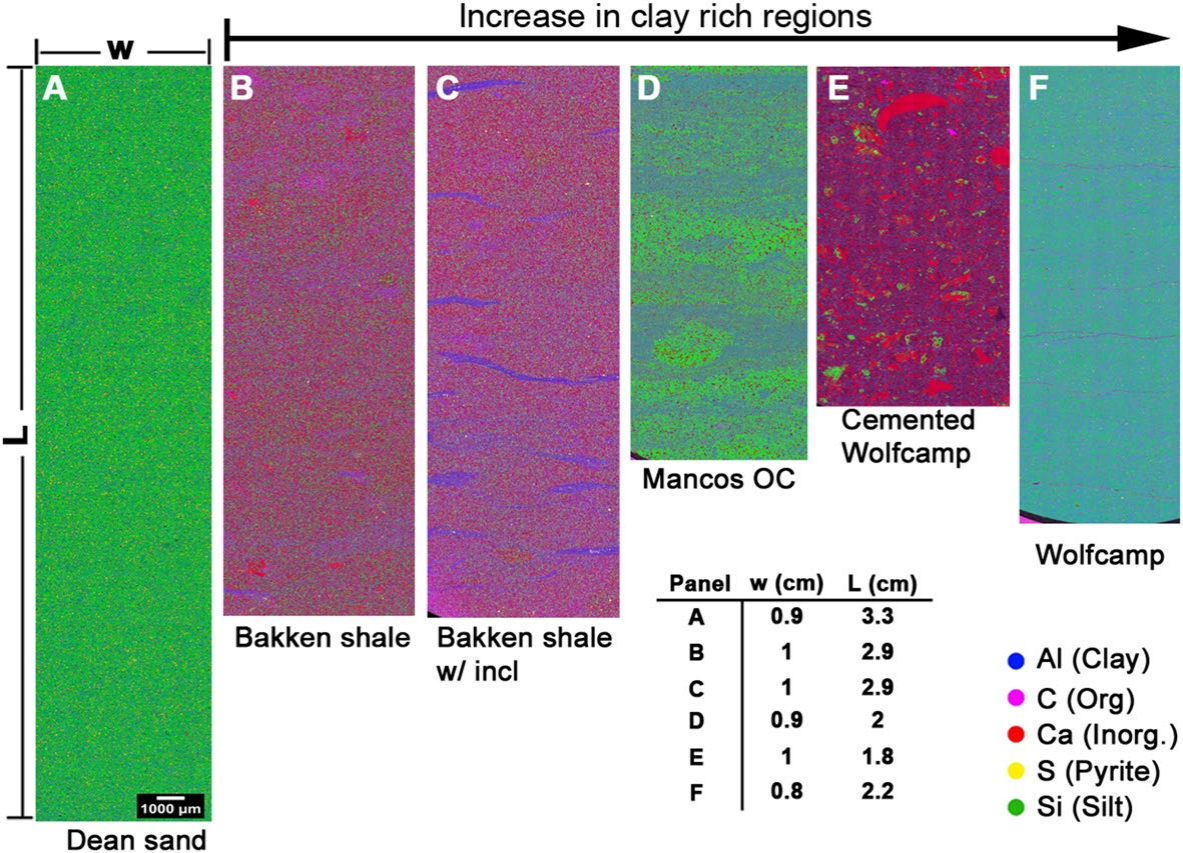
Areal electron probe microanalysis (EPMA) scans of various North American tight rocks with the Dean sandstone sample (**A**) shown for comparison. The tight samples were characterized for pressure dependent porosity and permeability. The samples are arranged in order of increasing clay content from left to right (also see Table 1). The legend shows colors corresponding to the predominant elements (and the associated mineral proxy in parentheses) which are used in a weighted sum dictated by the elemental mass fraction to determine the pixel color. Both Wolfcamp samples (**E**,**F**) have bedding parallel delamination cracks, but the cracks in the cemented Wolfcamp sample are barely perceptible. For this sample one crack emanates a few pixels below the top left corner of the elemental map, and the other is located about 0.4 cm from the bottom of the map.

Results for the EPMA scans on the samples studied in this work are shown in Fig. 1. Each panel is an image stack of the common elements found in unconventional reservoir materials which are commonly referred to as mudstones (e.g., Wolfcamp) and grainstones (e.g., Bakken, layers in the Mancos OC). In particular, the pixel color represents a weighted sum of the colors corresponding to the mass fraction of the elements that are present. These images are useful for inferring both the composition and microstructural morphology at larger length scale (~ cm). Figure 1 also shows the elemental map for a Dean sandstone to draw a contrast to more conventional reservoir materials that contain very little clay. The samples studied in this work consist of a greater proportion of finer sediment in the form of clay and silt (fine sand). The elemental maps in Fig. 1 are arranged in order of increasing proportion of clay content from left to right (Fig. 1). In addition, we have samples taken from the same well at depths that vary by no more than 100 ft in the Bakken and Wolfcamp shales. The Bakken shales are sedimentary carbonate grainstones. Both samples show very consistent background structure, however one of the samples has lenticular clay rich inclusions likely formed by bioturbation. These clay rich structures are nearly imperceptible to the naked eye. Both Wolfcamp samples have the same background composition of clay and silt, but one of them contains abundant carbonate fragments. These fragments appear to lend themselves to the uniform through-solution deposition of carbonate cement during diagenesis. Additionally, both Wolfcamp samples exhibit micro-scale bedding parallel delamination cracks, but these are much less abundant in the cemented analogue.

**Figure 2:**
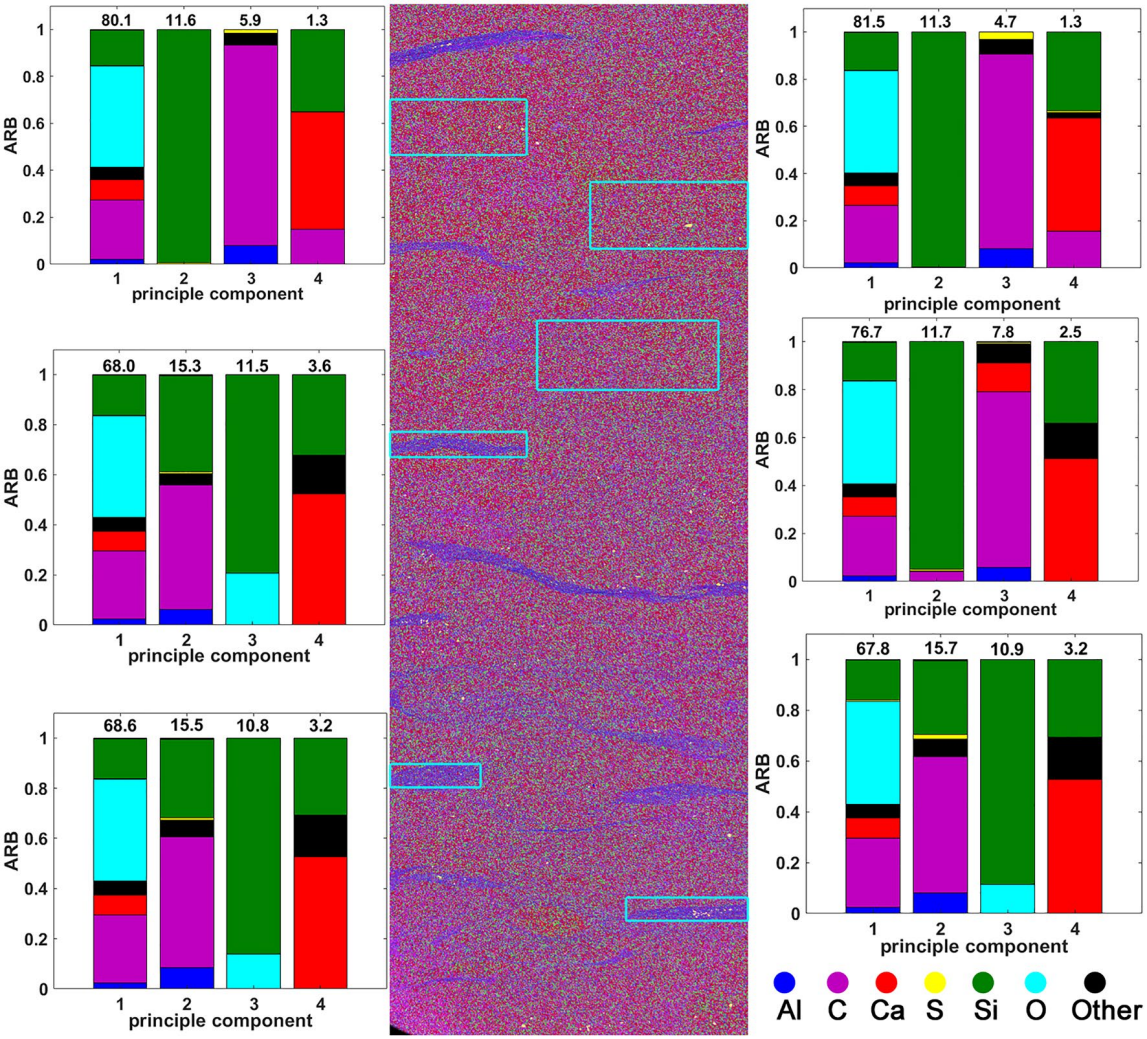
EPMA results for Bakken shale with lenticular clay rich inclusions. The panels on either side of the elemental map show the principal component analysis (PCA) for the adjacent image section outlined with a cyan rectangle. The PCA results show that the clay rich inclusions consist of a higher weighting of the principal component with elevated Al content.

**Figure 3:**
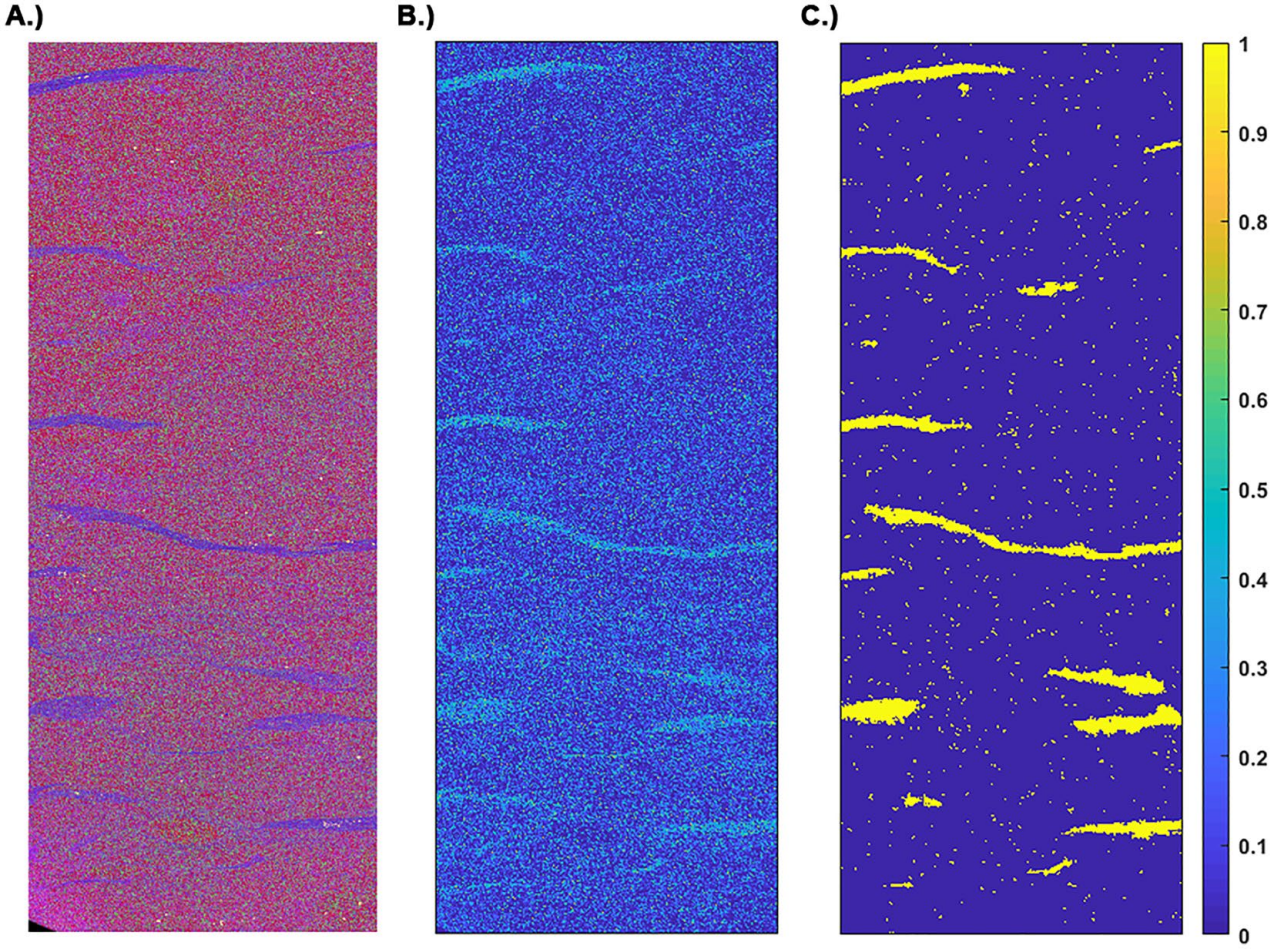
From left to right, (**A**) elemental map of Bakken shale with lenticular clay-rich inclusions, (**B**) normalized mass fraction of elemental Al only, and (**C**) binary image created using the Matlab image segmentation toolbox on the Al map. We determine that 5.7% of the area is occupied by the clay (Al) rich zones with the binary image (**C**).

Figure 2 shows the results of a principal component analysis (PCA) on the bioturbated Bakken shale. The PCA shows that the lenticular inclusions are characterized by an increase in the proportion of the principal component enriched in Al, the proxy for clay. In addition to indicating the microstructural morphology, the EPMA is useful for quantifying the volume fraction of each microstructural constituent, assuming consistency with the area fraction. The Bakken sample (Fig. 3A) primarily consists of grain supported carbonate background. As indicated in Fig. 3B the clay rich regions are characterized by an increase in Al content. To quantify the area fraction attributable to the clay rich regions we analyze the Al elemental map with the graph cutting routine in the Matlab image analysis toolbox. This routine utilizes a common image segmentation algorithm^31^ to produce the binary image (Fig. 3C). By simply summing all the yellow pixels and dividing by the total number of pixels in the binary image, we determine that the clay rich regions occupy 5.7% of the area. We repeat similar segmentation routines for all the samples except the uniform Bakken shale. The volume fraction of the main microstructural constituents in each sample is outlined in Table 1. These results demonstrate that the suite of samples utilized in this work, provide the ability to assess the effect of increasing clay content on both PDP, and PDPo.

**Table 1:** Volume fraction of microstructural constituents assumed to be equivalent to the area fraction inferred from the elemental maps after image segmentation like that shown in Fig. 3. Also shown are the values for the indentation modulus measured during nanoindentation for each constituent. The values in parenthesis are inferred by accounting for the microcrack compliance as discussed in the supplementary information (see Microcrack stiffness section, Eq. S9).

Sample	Volume fraction of relevant microstructural constituents					
	Clay	Cement clay	Quartz grain supported	Calcite grain supported	Calcite inclusions	Microcrack
Bakken	–	–	–	1	–	–
Bakken w/inclusions	0.057	–	–	0.943	–	–
Mancos	0.65	–	0.35	–	–	–
Wolfcamp cemented	–	0.77	–	–	0.23	(0.015)
Wolfcamp	0.98	–	–	–	–	0.02
	Compliance, *C*_P_ or *C*_S_ (GPa^−1^)					
Nanoindentation	0.04 ± 0.013	0.015 ± 0.002	0.017 ± 0.002	0.021 ± 0.04	0.014 ± 0.002	0.089 ± 0.01
Literature	0.05 ± 0.013^32^		*C*_P_ > *C*_S_ = 0.027^34^	*C*_P_ > *C*_S_ = 0.014^34^	*C*_S_ = 0.014^34^	
Crack mechanics						(23)

### Nanoindentation

**Figure 4:**
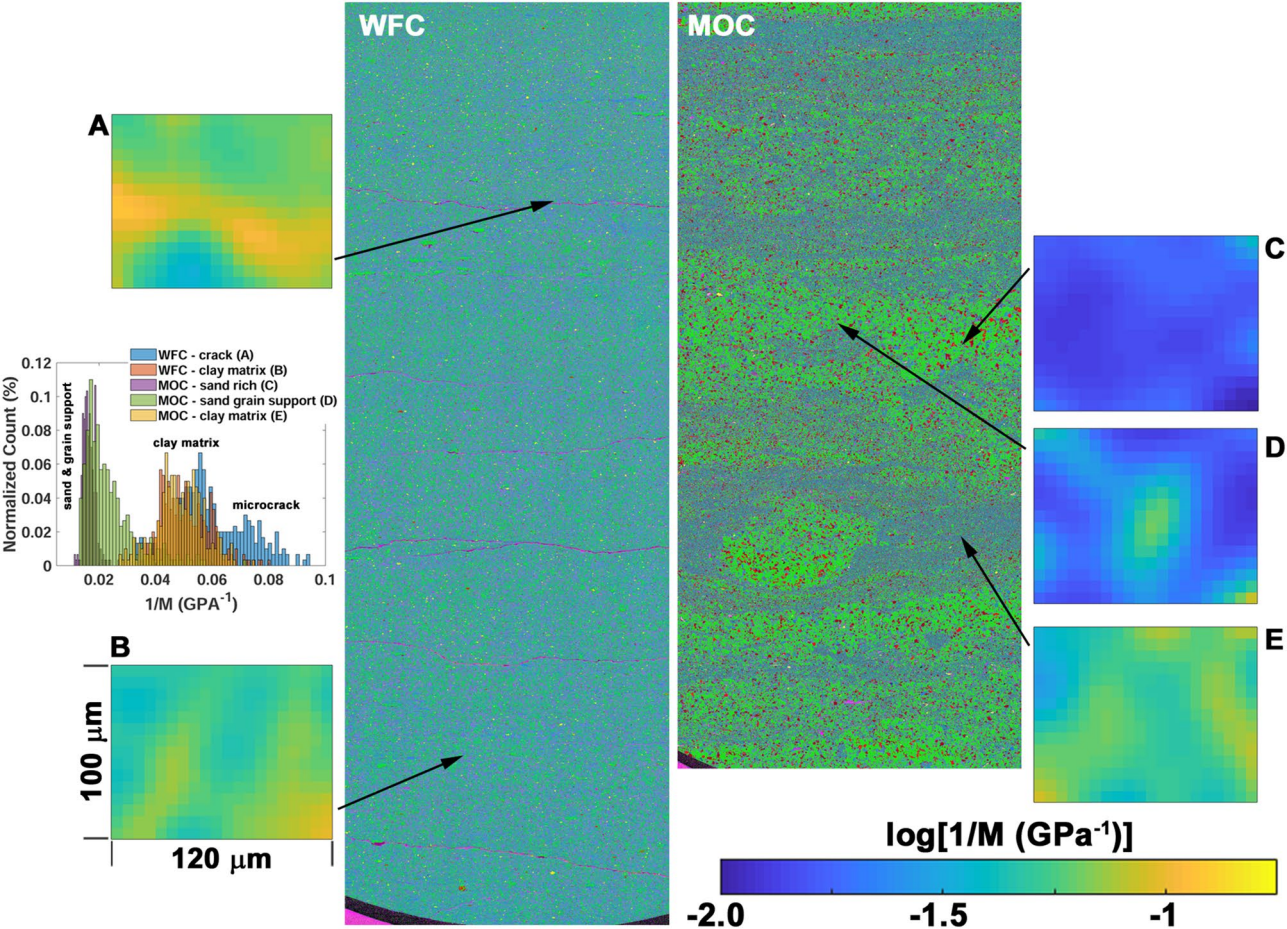
Nanoindentation maps (map area ~ 100 µm × 120 µm) measured on the Wolfcamp (WFC) and Mancos OC (MOC) samples. The measurements were performed on each of the common microstructural constituents encountered in these rocks: argillaceous clay rich regions (map **B**,**E**), grain supported (map **C**,**D**), and microcrack (map **A**). On the left, indentation map A clearly shows the measurement is sensitive to the presence of the microcrack. On the right indentation maps C (top) and D (middle) show the sensitivity to the silica grains through a reduced compliance (1/M). The histogram quantifies the nanoindentation statistics for each sample, where the letter in the legend corresponds to the labeled indentation maps. The clay rich regions show good consistency as clearly indicated by the log(1/M) distribution (corresponding to map B and E in the histograms). The histograms also indicate that the clay rich compliance extrema are good thresholds for isolating measurements on grain supported regions (1/M < 0.03 GPa^−1^, e.g., map **C**,**D**), or those areas proximal to the microcrack (1/M > 0.055 GPa^−1^, e.g., map **A**).

Figure 4 shows nanoindentation results from the Mancos OC and Wolfcamp sample with representative results for each type of microstructural constituent found in these rocks (Fig. 1). The indentation maps are smoothed with a Gaussian filter to suppress noise. Smoothing the image accentuates the features we target from the elemental maps like the microcracks (Fig. 4A, Supplementary Information—Fig. S5) or grain supported regions (Fig. 4C,D, Supplementary Information—Fig. S6). We note, that for the argillaceous clay rich regions (Fig. 4B,E, Supplementary Information—Figs. S5 and S6), we observe very similar effective compliance (Table 1), and indentation statistics, to that previously reported^32^. Moreover, the smoothing procedure has very little effect on the average compliance determined for each microstructural constituent. Therefore, we have confidence that the compliance inferred from smoothed nanoindentaiton maps offers a reasonable comparison to our analysis of the mechanical response.

The average values of the indentation compliance inferred from the maps taken for each microstructural constituent are shown at the bottom of Table 1. In the grain supported region (Fig. 4C,D), or in the vicinity of a microcrack (Fig. 4A) we isolate respective features by thresholding the indentation map. The threshold is determined from the average clay rich indentation modulus plus or minus one standard deviation. The histograms, from the raw indentation modulus (Fig. 4, Supplementary information—Fig. S7), show a comparison of the 1/*M* (≈ 1/(1.5*K*_P_) = 0.67*C*_P_) distribution for the maps from the Wolfcamp and Mancos OC samples. In the case of the microcrack (Fig. 4A) or the silica grains (Fig. 4C,D) the histograms indicate that the threshold is sufficient to isolate the response from the individual microstructural features. The inferred modulus of the calcite inclusions agrees very well with that found in the literature^33,34^ (Table 1). In the case of measurements on sand grains, or sand supported regions, the effective modulus is greater than that found in the literature^34,35^. This may be due to slight calcite cementation in the sand supported regions. In a similar manner the stiffness of the calcite cemented Wolfcamp clay matrix is much higher than that of the argillaceous counterpart. In fact, the inferred values are nearly identical to that measured on the calcite inclusion, and they are higher than that measured on the carbonate grain supported Bakken shale.

### Pressure dependent porosity and permeability

**Figure 5:**
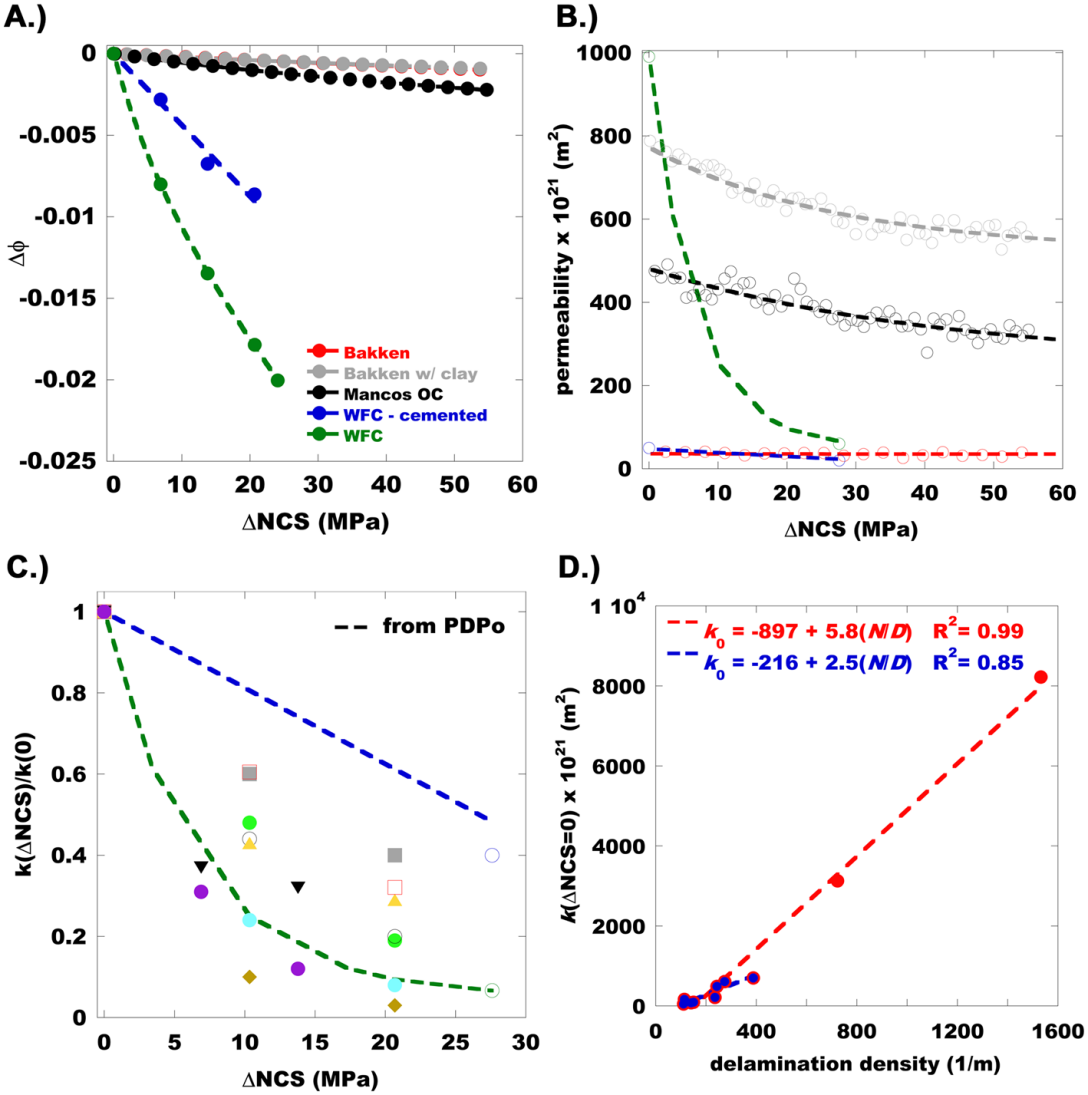
(**A**) Pressure dependent porosity (Δϕ) for five samples utilized in this study (Fig. 1), indicating a broad spectrum in response over a representative change in net confining stress (ΔNCS). (**B**) Simultaneous pressure dependent permeability measurements for the same samples as shown in (**A**). (**C**) Normalized pressure dependent permeability of clay rich mudstones with a priori response for Wolfcamp mudstones shown in (**A**). (**D**) Low net-confining stress permeability (*k*_0_) versus the delamination density inferred from the EPMA results. The dashed lines represent linear fits to Eq. (6) with a constant offset term.

The PDP and PDPo results are shown in Fig. 5. As anticipated from the nanoindentation results (Table 1) the sample compliance increases with clay content. The similar response of the Bakken samples demonstrates the strong effect of grain support when the more compliant clay rich inclusions are isolated. In fact, the PDPo results contradict the nanoindentation which indicates the Bakken shale should be more compliant than the carbonated wolfcamp sample. This observation certainly casts doubt on our ability to universally constrain the first term in Eq. (2) with the indentation results.

Figure 5A shows that we are able to obtain excellent agreement between the PDPo data and Eq. (2) with three free parameters (*C*_S_-*C*_P∞_), *ϕ*_Slit_, and ∂*C*_P_/∂*ϕ*_Slit_ (Table 2). This approach does not guarantee that we extract a unique set of parameters under the constraint of minimizing the difference between the data and Eq. (2). As a result, we compare the extracted parameters to measured or inferred values (e.g., *ϕ*_Slit_ < *ϕ*, *C*_S_, *C*_P_) to gain confidence in our results (Table 2). This comparison shows that the inferred compliance, *C*_P∞_, for the samples with grain support are in much better agreement with the nanoindentation/EPMA informed EMT predictions (Eq. (3)), than that observed for the clay rich Wolfcamp mudstones. Both mudstones contain delamination cracks, and the nanoindentation measurements on the siliceous Wolfcamp sample indicate that (Fig. 4A) the highest compliance is observed in the vicinity of the microcrack. However, this simply implies the crack, and surrounding material, are more compliant. The crack compliance is governed by the contact area, which is related to the surface topography. Given the crack surface topography measured on a companion Wolfcamp sample, the crack compliance,* C*_P_ = 23 GPa^−1^ (Table 1), may be predicted when the stress is oriented perpendicular to the crack plane^36–38^ (See supplementary information—Microcrack stiffness). As shown in Table 2 the compliant microcracks account for the large compliance exhibited by the argillaceous Wolfcamp sample during the PDPo measurements. Since the microcracks in the cemented Wolfcamp sample are indistinguishable from the background, we take a similar approach but in this case we infer the volume fraction of microcracks, which is shown in Table 1. As expected from the EPMA results, this value is smaller than that determined for the argillaceous analogue, and within the error associated with inferring the volume fractions from the EPMA maps through image analysis (e.g., see background speckle in Fig. 3C, Fig. S4—crack px only). From this analysis it is clear that the high inferred *C*_P∞_ of the Wolfcamp mudstones is due to the compliant microcracks. The compliance of these features is 3 orders of magnitude greater than that inferred from nanoindentation (Table 1). It is also worth noting that, consistent with the contrast in PDP response, the delaminated argillaceous mudstone shows the most severe non-linearity, as quantified by the parameter, ∂*C*_P_/∂*ϕ*_Slit_ (Table 2), and those samples with concentrated clay rich regions show an order of magnitude less non-linearity.

**Table 2:** Parameters extracted from the pressure dependent porosity (PDPo) and permeability (PDP) data shown in Fig. 5. Also shown is a comparison of the inferred *C*_P∞_ to that expected from Eq. (3), *C*_P,EMT_. Finally, the inferred pore sizes, *r*_P_ are compared to that measured via common microstructural characterization techniques. The value in parenthesis is inferre﻿d by accounting for the microcrack compliance as discussed in the supplementary information (see Microcrack Stiffness section, Eq. S9).

Sample					EMT	Poro	MICP/N_2_		Poro
	*C*_S_-*C*_P∞_ (GPa^−1^)	*ϕ*_Slit_	d*C*_P_/d*ϕ* (GPa^−1^)	*C*_P∞_ (GPa^−1^)	*C*_P,EMT_ (GPa^−1^)	2*r*_P_ (nm)	2*r*_P,meas_ (nm)	*ϕ*_*meas*_	*b* (nm)
Bakken	− 0.014	–	–	0.028	0.032	4.4	13	0.035	
Bakken w/ inclusions	− 0.013	0.0002	0.038	0.027	0.033	16.4	12	0.048	6210
Mancos	− 0.018	0.0017	0.023	0.045	0.044	8.8	5	0.068	1840
Wolfcamp cemented	− 0.44	–	–	0.43	0.022	6.4	7.3	0.025	
Wolfcamp	− 0.58	0.006	0.154	0.55	0.06 (0.5)	–	3.3	0.11	1160

The mechanical and microstructural parameters inferred from the PDPo and EPMA results are utilized to analyze the complimentary permeability measurements shown in Fig, 5B. In the case of the Wolfcamp samples there are only two permeability measurements because establishing a steady state takes several weeks when the pore fluid is a liquid. As a result, an a priori prediction is afforded by substituting *ϕ*_0_ − Δ*ϕ* (Eq. (2)) into Eq. (4). The change in porosity is weighted by the volume fraction of the constituents where the porosity is predominantly slit porosity in the clay (Table 1). Otherwise, we simply fit Eq. (4) to the data. In both cases we ensure agreement with the low *p*_CS_ permeability *k*(Δ*p*_CS_ = 0) by solving Eq. (4) to obtain a relationship between *r*_P_ and *b*, given *ϕ* and *ϕ*_Slit_ inferred from the PDPo data. In the case of the argillaceous Wolfcamp sample, the measured value of *r*_P_ is utilized to constrain this prediction. We note that the fits and a priori predictions exhibit excellent agreement with the data. In all cases except the argillaceous Wolfcamp sample we extract an estimate of *r*_P_ from the fitting parameters, then use the Δ*p*_CS_ = 0 constraint to determine *b* given *r*_P_. Since the microstructural characterization techniques were performed on granulated (~ mm particles) or pulverized sample (100 μm particles), we are only able to make a comparison to *r*_P_. There is reasonable agreement between the inferred *r*_P_ and the characteristic value inferred from the complimentary measurements (Table 2) with the greatest discrepancy observed for the Bakken shale. In this case, *r*_P_ is inferred from a permeability measurement on a cm scale core, while the pore size was measured on mm sized granules; This suggests that while these larger pores are present, they do not control mass transport. In other words, at the two different length scales access to the porosity is afforded by slightly different pore sizes. It is also worth mentioning that at the highest pressure employed during the measurement, mercury is not capable of accessing pores with 2*r*_P_ < 5 nm. Recall that we assumed the tortuosity in the expression for *k*, χ ≈ 5–12 (Eq. (4)), which is consistent with recent work for bedding parallel flow^10^. The microstructural characterization techniques also demonstrate that carbonation, and grain support, result in lower porosity (Table 2).

The Wolfcamp samples exhibit the most severe PDPo and PDP behavior, and analysis of the PDPo data indicates this is due to the delamination cracks parallel to the bedding direction (Fig. 1). Figure 5C shows PDP normalized by *k*(Δ*p*_CS_ = 0) from a collection of argillaceous mudstones and the *a prior* response for the Wolfcamp samples considered heretofore. The a priori predictions bound the response envelop for these types of rocks. We note that those samples near the upper bound exhibit a roughly linear response, and the non-linearity increases with proximity to the lower bound, or as the pressure dependence becomes more severe.

**Figure 6:**
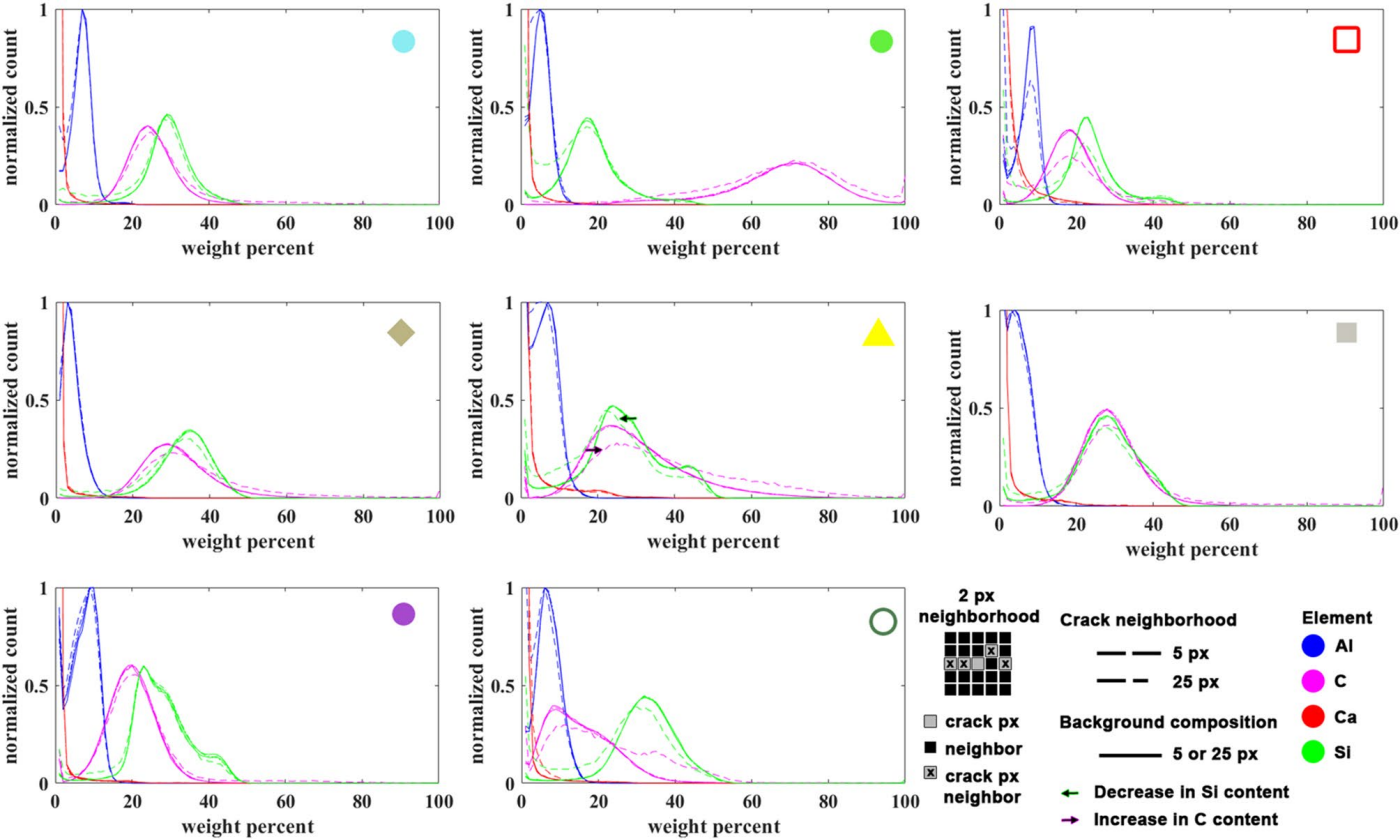
Histograms quantifying the elemental composition in the vicinity of the cracks for a 5 px (25 µm) or 25 px (125 µm) neighborhood. Schematic in legend demonstrates how the histograms are calculated for a 2 px neighborhood. Also shown is the background elemental composition over the same length scale. As indicated by the highlighted arrows in the central panel the delamination cracks in most of these rocks tend to seek out trajectories that are enriched in organic carbon, offset by a similar reduction in silica content. The subtle compositional shift extends at least 25 µm but less than 125 µm from the crack. Symbols in the upper right corner of each panel correspond to the sample shown in Fig. 5C.

The samples that contributed to the data in Fig. 5C were also characterized by EPMA. Every one of these samples contained delamination cracks like that found in the Wolfcamp rocks (Fig. 1). Figure 5D shows *k*(Δ*p*_CS_ = 0) as a function of the delamination density determined from the EPMA maps (see supplementary information—Delamination density, and Fig. S4). As expected from Eq. (6), we find the low *p*_CS_ permeability exhibits a linear dependence on the delamination density (*N*/*D*). Given that ~ 20% of the samples exhibit uniquely high *k*(Δ*p*_CS_ = 0) we fit Eq. (6) to all the data (Fig. 5D—red dashed line) and to those data characterized by *k*(Δ*p*_CS_ = 0) < 10^–18^ m^2^. It is worth noting, that the latter subset exhibits a range in *k* that extends over an order of magnitude. Owing to the strong dependence on the crack aperture the difference in average delamination aperture determined from the two slopes (Eq. (6)) yields the range, 290 < *b*(nm) < 380. We note that this aperture is consistent with that shown in the scanning electron micrograph of the indentation map shown in the supplementary information Fig. S5. Comparing these values to that reported for the siliceous Wolfcamp sample in Table 2, *b* = 1160 nm, suggests that 3–4 delamination cracks are responsible for the increase in *k*(Δ*p*_CS_ = 0). This is roughly consistent with the number of delaminations observed on the elemental map (Fig. 1 ~ 6 cracks), which nearly spans the sample diameter. In addition, the linear fits to Eq. (6) shown in Fig. 5D may be set equal to zero to determine a critical delamination density for permeability enhancement, *k*(Δ*p*_CS_ = 0) = 0, 85 < *N*_*C*_/*D* (m^−1^) < 155. Therefore, when the average delamination spacing is greater than ~ 1 cm, like that observed for the cemented Wolfcamp sample, the associated permeability enhancement is negligible. Finally, with each crack pixel identified, we compare the elemental composition of the sample (Fig. 6—Background) to that in the neighborhood of the cracks for the samples utilized to generate Fig. 5C. This analysis indicates that the cracks tend to seek trajectories in zones that are slightly enriched with organic carbon, corresponding to a slight decrease in silica content (Fig. 6). The subtle compositional shift extends at least 25 µm, but no more than 125 µm from the crack.

The Mancos (which is an outcrop sample) and bioturbated Bakken do not have delamination cracks. The inferred values of *b* are indicative of the role grain support plays in preserving larger interstitial clay pores. In fact, the pore size distribution (Supplementary information, Fig. S3) of the grain supported rocks is shifted to larger pore sizes. Grain support shields interstitial clay from the increasing compressive loads experienced as the overburden increases. Therefore, there is no impetus for the interstitial clay to seek an intimate aligned packing configuration, and larger pores are preserved. In fact, these samples exhibit a relatively high permeability over the entire range of *p*_CS_ investigated when compared to the Wolfcamp or homogeneous Bakken samples. Pursuing this comparison further leads one to conclude that the interstitial clay dominates transport at high *p*_CS_. This must be the case because the clay rich Wolfcamp samples exhibit much lower permeability at high *p*_CS_. For these rocks, the delamination cracks contribute to the large PDPo response, but the high *p*_CS_ permeability is both consistent and significantly lower than that of the Mancos or bioturbated Bakken.

## Conclusions

Unconventional reservoir materials exhibit a broad spectrum of pressure dependent porosity and permeability. This work demonstrates that the breadth of this mechanical response may be attributed to a few microstructural constituents that are found in most tight rocks. Argillaceous tight rocks exhibit the most severe, non-linear, pressure dependence because they are susceptible to imperceptible bedding parallel microcracks. These delamination cracks form in planes that are slightly enriched with organic carbon. The low net confining stress permeability of argillaceous tight rocks is linearly proportional to the microcrack density. At low net confining stress the crack aperture is roughly two orders of magnitude larger than the pore size controlling transport in the rock matrix. These cracks have a critical density value that corresponds to an average crack spacing of roughly 1 cm. At lower density (larger spacing) the effect on permeability is negligible. In the absence of these cracks clay rich regions exhibit significantly less non-linear pressure dependence. Providing grain support shields the interstitial clay from stress, reducing the stress dependence even further and eliminating the non-linearity altogether. These new insights are pertinent because the argillaceous mudstones have the highest porosity, and thus represent the highest resource potential.

In addition, this work demonstrates that complimentary pressure dependent porosity measurements are necessary to account for the contribution of each microstructural constituent to the overall magnitude and linearity of permeability degradation. In the absence of these measurements an estimate may be made with effective medium theories informed by the volume fraction of constituents (e.g. from elemental maps), and the spatially constrained mechanical properties (e.g., nano/micro-indentation). It is worth noting that for most of the constituents encountered in shale or tight grainstones, we report compliances that are consistent with those found in the literature. Cracks exhibit unique highly non-linear mechanical properties that are not amenable to experimental characterization. Therefore, when cracks are present it is not possible to fully inform models that account for pressure dependence with spatially constrained mechanical property measurements. In this case, an estimate of the mechanical properties is provided from the crack surface topography and previously derived theories for the crack compliance. To account for the degree of non-linearity this work provides an indication of representative values for cracks, and clay-rich constituents, which differ by an order of magnitude. Once, the pressure dependent porosity is characterized or predicted, the effective permeability reduction is given by the weighted porosity reduction in a superposition of commonly employed permeability models for the pore architectures.

Conventional hydraulic fracturing techniques create large bi-wing vertical fractures (perpendicular to bedding) that are propped with sand (~ 100 µm). This technology is not optimized to exploit the indigenous weak bedding parallel planes (**O**(*b*) ~ 10^–1^ µm) that lend themselves to large increases in the effective permeability of oil-bearing rock. Therefore, the findings discussed heretofore may be utilized to devise complimentary well completion technologies to exploit, then circumvent pressure dependence in practice. Moreover, effectively exploiting these indigenous weaknesses should provide access to more pores adjacent to the primary hydraulic fracture, ultimately improving recovery factors. The associated increase in effective permeability should also accelerate oil production, reducing the production overhead. Improving recovery factors and reducing production overhead will ultimately reduce the carbon footprint of oil produced from unconventional oil and gas assets.

Finally, core scale (~ cm) observations may be integrated into models to account for rock-specific pressure dependent permeability at the reservoir scale. These models aim to quantify fluid drainage into large (~ 200 m) bi-wing hydraulic fractures at lower pore pressure. The spatiotemporal fluid flow, associated pore pressure, and net confining stress are discretized over a grid with characteristic scale on the order of ~ m that spans half the rock volume ~ 10 m between adjacent bi-wing fractures. Therefore, accurate determination of the temporal change in the net confining stress field requires representative mechanical and transport properties at each point on the grid. The work presented here may assist in populating the model with these properties given the composition, microstructural morphology, microstructural constituent mechanical properties, and delamination density for a representative spectrum of rocks encountered in the basin (e.g.: Tables 1 and 2). It may be necessary to use the techniques employed here to inform effective medium theories for upscaling from the core to the grid scale. When the reservoir model is populated with representative material properties it is straightforward to account for pressure dependent permeability at each time step given the spatial variation in net confining stress (e.g., using Eqs. 2 and 4). Upscaling core scale measurements to the grid, then reservoir scale, may introduce large errors especially if the model implementation is informed by sparse complimentary measurements like rock composition inferred from well logs. As a result, relevant parameters like rock composition, microstructural constituent mechanical properties, and delamination density may be stochastically varied to yield a range of model predictions. The spectrum of deterministic model outcomes may be informed by the variance in measurements corresponding to a broader scale, such as rock composition throughout an entire basin. Alternatively, variance in parameters like microstructural constituent mechanical properties, or delamination density may be inferred from experiments on representative core. As previously noted, conventional hydraulic fracturing creates large bi-wing vertical fractures, and thus may not uniformly enhance permeability throughout the intervening rock. In this case, an additional free parameter is necessary to characterize the length scale of permeability enhancement, and thus pressure dependence, in the rock adjacent to the bi-wing fracture. For instance, the length scale for permeability enhancement in argillaceous mudstones could be constrained by theories or experiments to characterize the delamination correlation length.

## Supplementary Information

Supplementary Information.

## Data Availability

The raw data are not publicly available due to company policy. The raw data that support the findings of this study are available upon reasonable request from the corresponding authors J.J.V., subject to approval from ExxonMobil.
