# Supplementary material for: Origins of pressure dependent permeability in unconventional hydrocarbon reservoirs
Source: Sci Rep. 2023 May 2;13:7111. doi: 10.1038/s41598-023-33601-5 (PMC10154339; doi:10.1038/s41598-023-33601-5)
Supplement: Supplementary file 1 — Supplementary Information. [file 41598_2023_33601_MOESM1_ESM.pdf]

Supplementary information

Methods

Sample preparation for EPMA and Nanoindentation

Prior to measuring the elemental composition with the microprobe a ~1 cm thick disc was removed from each core. One face of this disc was polished with a Buehler EcoMet 250 under 4-lbs of compressive force. Initially the samples were coarse polished with diamond embedded abrasive sheets in n-heptane. The abrasive grit was sequentially increased from 180 grit (78 μm) to 1100 grit (8 μm). The polished surfaces were finished with Buehler TriDent polishing cloth using 6, 3, then 1 μm diamond sprays (in alcohol). Finally to prevent charging from the electron beam, the sample was carbon coated under vacuum. We estimate that the carbon coating is about ~20 nm thick.

Electron Probe Microanalysis experimental details

The spot size of the electron beam is on the order of 1 μm, while the pixel size for each image is 4.97 μm. The difference between the spot and pixel size reflects the fact that the electrons scatter in the near surface volume. Therefore, the electrons interact with atoms not immediately under the illuminated spot. The final measurement consists of an image that is up to 1 cm wide by 3.5 cm high. To interrogate such a large area we stitch together measurements on smaller areas (256 × 190 pixels). The dwell time on each pixel is 50 μs, and each area is scanned 125 times to permit averaging.

Notes

Poromechanical response in the low frequency regime

The compliance of the porosity, ϕ, is given by:

$$\frac{d\phi}{dp_{CS}} = C_S + C_P\left(1-\phi\right) \qquad \text{(S-1)}$$

All that remains is to account for changes in C_P associated with changes in ϕ, which can be estimated from a Taylor series expansion about the point, ϕ=0

$$C_P = C_{P\infty} + \frac{\partial C_P}{\partial \phi_{Mat}}\phi_{Mat} + \frac{\partial C_P}{\partial \phi_{Slit}}\phi_{Slit} \qquad \text{(S-2)}$$

where $C_{P\infty}$ is the high stress compliance of the rock consisting of the situation with all slit pores closed. In the limit $\phi\rightarrow 0$, $C_{P\infty}\rightarrow C_S^+$. In essence equation (S-2) describes the effect of *adding* porosity to a solid block with compliance C_S. Therefore, effective medium theories are useful for assessing the overall effect of adding the two types of pores. In particular previous work shows that the compliance of slit pores depends strongly on the pore aspect ratio, and thus leads to the realization [34]:

$$\frac{\partial C_P}{\partial \phi_{Mat}}\phi_{Mat} << \frac{\partial C_P}{\partial \phi_{Slit}}\phi_{Slit} \qquad \text{(S-3)}$$

Equation (S-3) is especially applicable at low p_{CS} (see microcrack stiffness section below). With this understanding little error is introduced by ignoring the second term on the right hand side of equation (S-2). If we substitute this simplified version of equation (S-2) into equation (S-1) we have

$$\frac{d\phi}{dp_{CS}} = C_S - C_{P\infty} - \frac{\partial C_P}{\partial \phi_{Slit}} \phi_{Slit} \qquad \text{(S-4)}$$

By inspection the compliance of the two types of porosity:

$$\begin{aligned} \frac{d\phi_{Mat}}{dp_{CS}} &= C_S - C_{P\infty} \\ \frac{d\phi_{Slit}}{dp_{CS}} &= -\frac{\partial C_P}{\partial \phi_{Slit}} \phi_{Slit} \end{aligned} \qquad \text{(S-5)}$$

Equations (S-5) indicates that as p_{CS} varies the deformation in the matrix porosity is due to the compression of the solid phase and that of the porous medium. On the other hand, as the pressure varies the deformation of the slit porosity is modulated by the change in the compliance associated with the increase in contact area throughout the slit, guaranteeing that the response is nonlinear. These ordinary differential equations may be integrated to determine an explicit dependence on pressure

$$\begin{aligned} \phi &= \phi_{Mat} + \phi_{Slit} = \phi_{Mat,0} + \left(C_S - C_{P\infty}\right) p_{CS} + \phi_{Slit,0} \exp\left(-\frac{\partial C_P}{\partial \phi_{Slit}} p_{CS}\right) \\ \Delta\phi &= \phi(p) - \phi(0) = \left(C_S - C_{P\infty}\right) p_{CS} - \phi_{Slit,0}\left[1 - \exp\left(-\frac{\partial C_P}{\partial \phi_{Slit}} p_{CS}\right)\right] \end{aligned} \qquad \text{(S-6)}$$

Microcrack stiffness

When two surfaces are pressed into contact, the relative displacement at a given load is governed by the strain energy associated with deformation at multiple points of contact. Ultimately the surface topography governs the contact area, and the contact area increases with the applied stress. As a result, the interfacial stiffness increases with applied stress. This is inherently what leads to non-linearity in classic contact mechanics [26]. A significant amount of recent work has focused on relating the imposed stress to a relative interfacial separation when the surface topography is known. The stress necessary to manipulate the crack aperture, b, is given by [36]

$$\sigma = 0.75\beta q_0 h_{RMS} E^* e^{-\alpha b/h_{RMS}} \qquad \text{(S-7)}$$

where the contact modulus is given by

$$\frac{1-\nu^2}{E^*} = \frac{2(1-\nu^2)}{E_P} \qquad \text{(S-8)}$$

and h_{RMS} is the root mean square roughness, q_0 is the smallest wave vector over which the surface is self-affine, and the constants α and β depend on the surface topography. For self-affine surfaces these constants depend on the Hurst exponent, H, which is related to the fractal dimension, H=3-D_f. The power spectrum of the spatial variation in height scales with the wave vector ~ q^{-2-2H} on self-affine surfaces [37]. **Fig. S-2** shows the fracture surface topography of an analogue argillaceous Wolfcamp mudstone, and the 2D and 1D power spectrum of this data. It is clear from the data that the topography is self-affine

over the entire range of wave vectors accessible during this experiment, and H=0.66. Therefore, previous work shows that α and β are **O**(1) [36]. In addition, the topography data indicates q_0=0.000035 μm^{-1} and h_{RMS}=12.4 μm. Therefore, equation (S-7) indicates the contact stiffness is less than $q_0 h_{RMS} E^*$=0.00054E^*≈0.00027K_P at incipient contact when b is ~ 3h_{RMS} while at moderate p_{CS} the $b \rightarrow h_{RMS}$ so the compliance of the crack is 2-3 orders of magnitude higher, ~0.002K_P, than the surrounding solid [38]. From this we infer the crack compliance is C_P ~ 23 GPa^{-1} (**Table 1**). We utilize this value in the exact effective medium theory for a layered medium with layer volume fraction ϕ_i, compliance, C_i, and i=1, 2:

$$C_{P\infty} = \phi_1 C_1 + \phi_2 C_2 \qquad \text{(S-9)}$$

to demonstrate the crack compliance accounts for the large $C_{P\infty}$ inferred from the PDPo measurements (**Table 2**). We take the same approach for the cemented Wolfcamp sample. However, in this case we use the effective modulus of the particulate laden clay rich cemented background as one compliance (e.g. C_1 ~ Eq. (3)), and the microcrack stiffness determined here as the other (C_2 ~ 23 GPa^{-1}). Since the microcracks are much less apparent in the EPMA map for this sample (**Fig. 1**), we infer the volume fraction of microcracks necessary to account for the compliance determined ($C_{P\infty}$) from the PDPo measurements (**Table 2**). This value is shown in parenthesis in **Table 1**.

Delamination density

The backscattered electron image for the argillaceous Wolfcamp sample is shown in **Fig. S- 4**. First the crack pixels are identified in a manner identical to that utilized to determine the volume fraction of the microstructural constituents (**Fig. S- 4**, crack px only). Then, we determine the distribution of distances between all crack pixel pairs. The delamination density is determined from the distance between nearest neighbors which we identify as the first maximum of the crack pixel spacing distribution. Since the microcracks are parallel to the bedding, we found that there was little difference between the absolute 2D delamination density and that determined by simply considering the 1D pair separation in the direction perpendicular to the cracks.

Figures

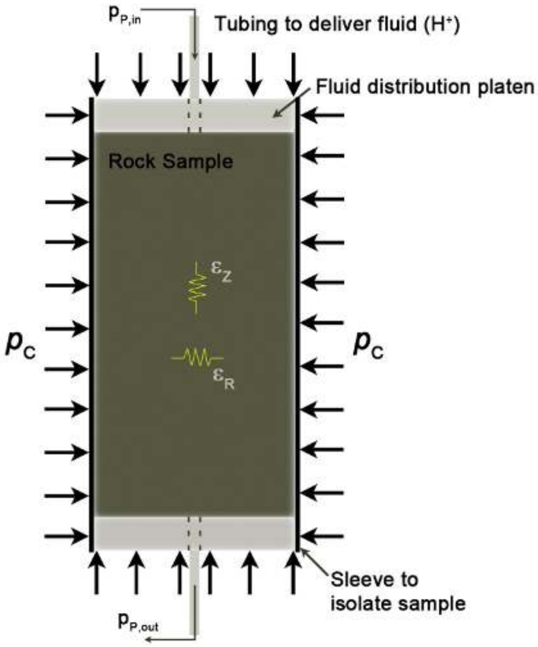

Fig. S-1. Schematic of hydrostatic pressure dependent porosity and permeability measurements. The change in porosity is assumed to be equivalent to the volumetric strain, as determined from the longitudinal (ε_Z) and circumferential strain (ε_R), with the mechanical testing technique. Alternatively, the change in porosity is given by changes in the proton count (H^+) with the NMR technique. All samples consisted of horizontal cores, so fluid flow is parallel to bedding.

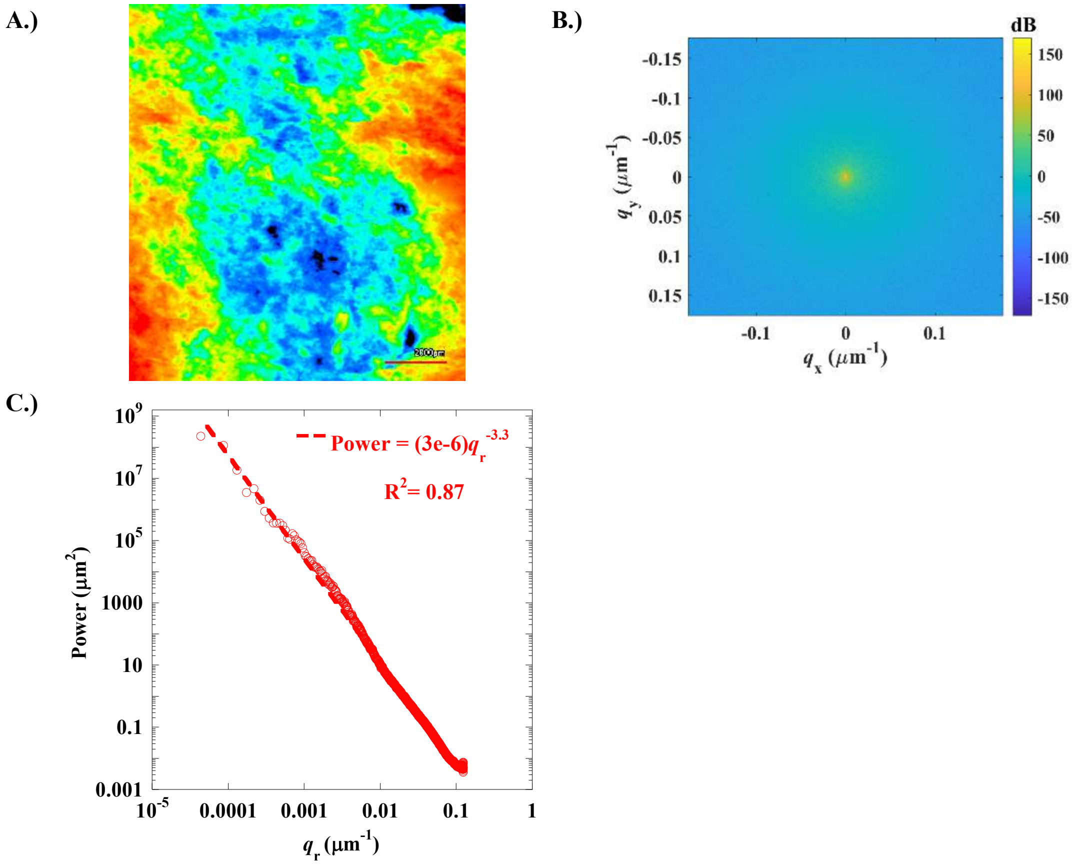

Fig. S-2. A.) Surface topography from analogue siliceous argillaceous Wolfcamp mudstone measured with scanning laser optical profilometry at a magnification of 5x. The scale bar represents a length of 2 mm. B.) 2D power spectral density of topography shown in A. C.) Average radial power spectral density from data shown in B. The power law fit shows that the surface is self-affine over several orders of magnitude in wave vector, and the exponent equals -2-2H, so the Hurst exponent is, H=0.66. The topographic map (A) is generated using ImageJ (v. 1.53c, http://imagej.nih.gov/ij).

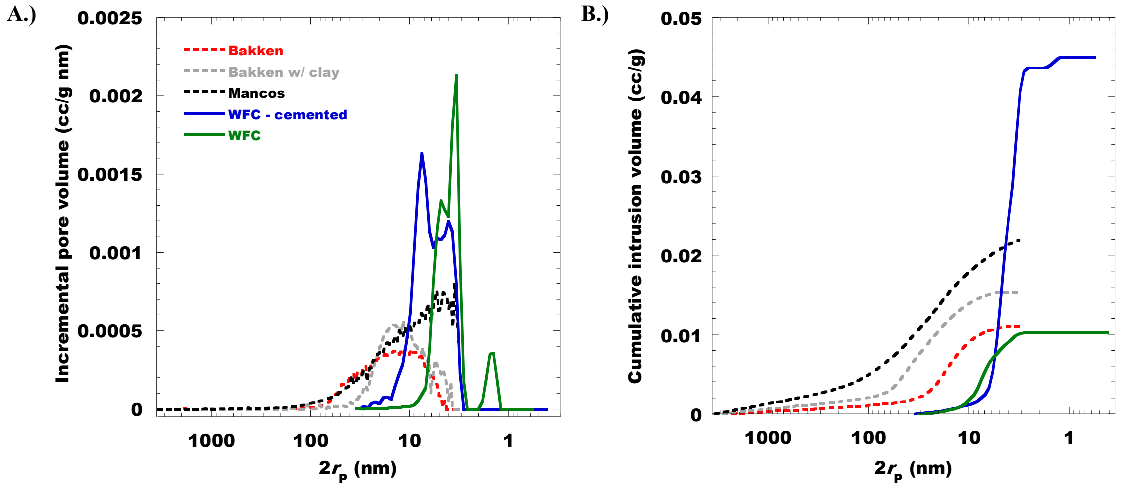

Fig. S- 3. A.) Incremental intrusion volume from mercury intrusion (dashed) or nitrogen desorption. A rough estimate of 2r_P is taken from the peak in the incremental pore size distribution. Note the small peak below 2r_P=2 nm for the Wolfcamp sample is due to cavitation during desorption. B.) Cumulative intrusion volume from the same measurements, the maximum intrusion volume is used to estimate the porosity assuming the bulk density of the rock is ~ 2.5 g/cc.

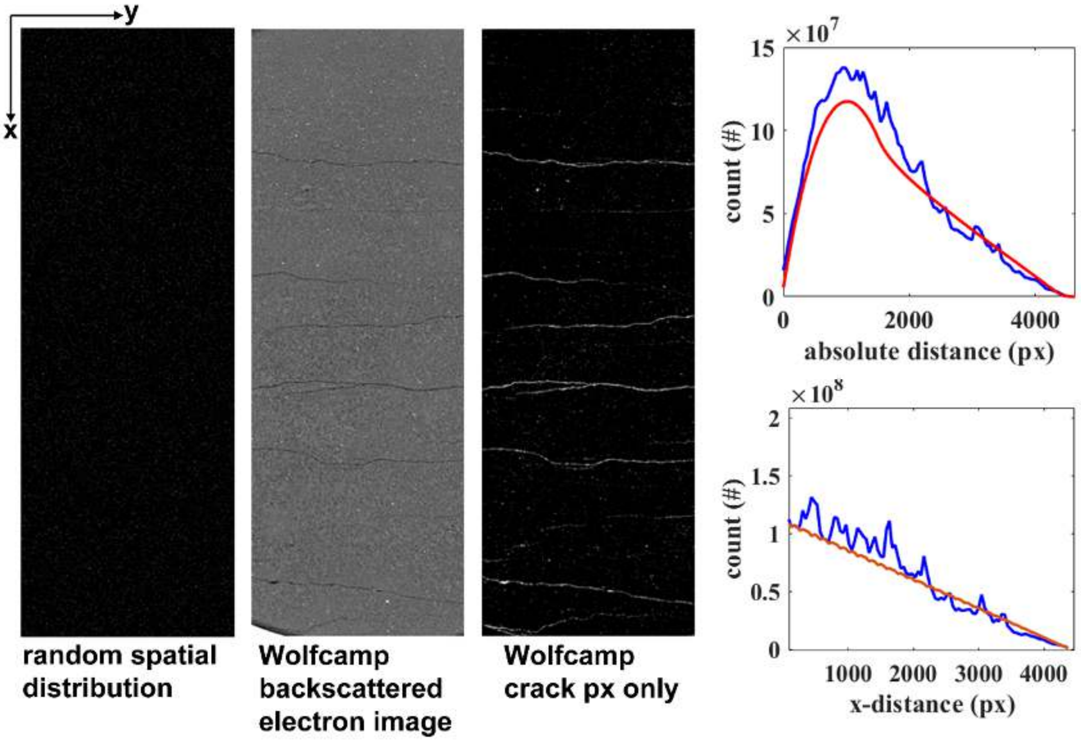

Fig. S- 4. Data used to quantify delamination density. Three grayscale rectangular panels on left show a random distribution of white pixels, the back scattered electron image from the EPMA measurement on the Wolfcamp sample, and the crack pixels isolated from the EPMA measurement. The two plots on the right hand side show the histogram for the absolute distance between the crack pixels, and the separation between the crack pixels in the x-direction only. Also shown in these plots is the same spacing for the random distribution of white pixels. The first peak in the x-spacing histogram is used to quantify the delamination density. In this case that peak is located at 441 px, or 0.219 cm, giving a delamination density of 456 m^{-1}. The intensity map (backscattered electron image) is generated using ImageJ (v. 1.53c, http://imagej.nih.gov/ij).

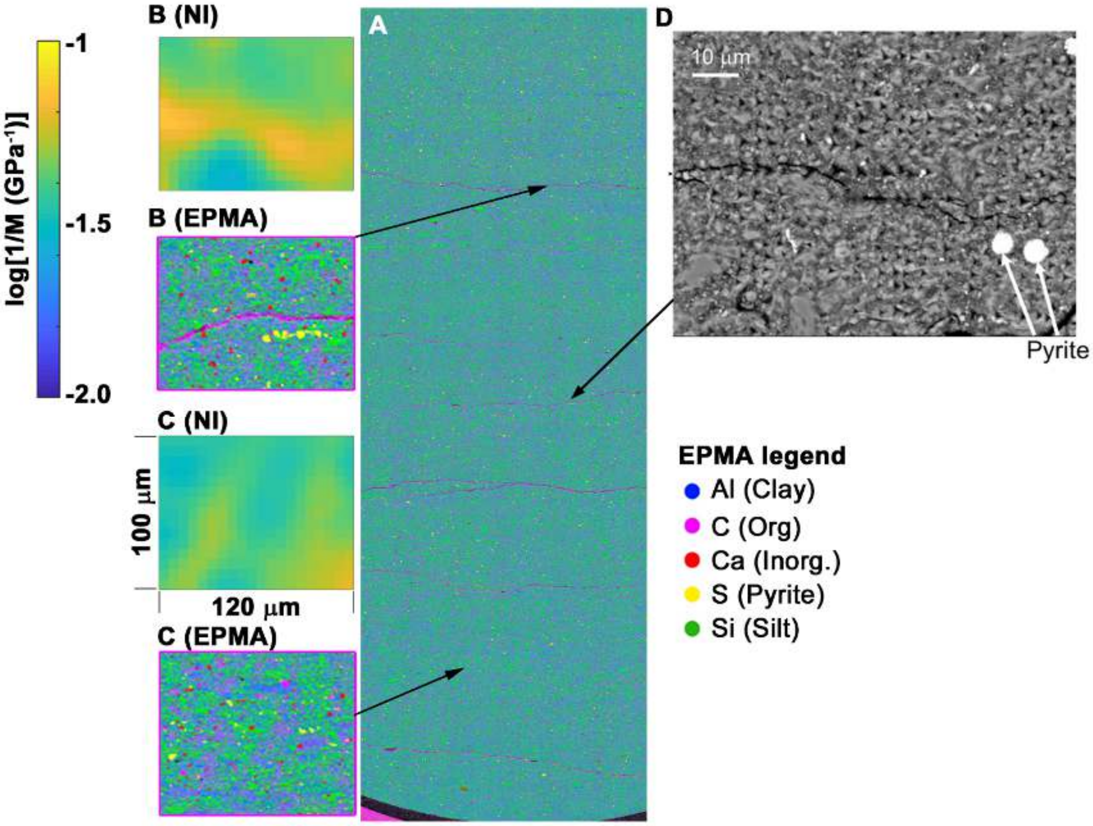

Fig. S- 5. Combined electron probe microanalysis and nanoindentation results for the Wolfcamp sample. A) Areal EPMA map reproduced from **Fig. 1**. B.) Nanoindentation map (NI) and magnified view of EPMA map (EPMA) for small area (120 µm x 100 µm) surrounding a delamination crack. C.) Nanoindentation map (NI) and magnified view of EPMA map for small area in argillaceous rock matrix. D.) Scanning electron microscope image of indentation map around delamination crack. Using the size of the indentation divot as a qualitative proxy for compliance indicates the area proximal to the crack is more compliant than that farther from the crack. The elemental map (A) is generated from the individual mass fractions using ImageJ (v. 1.53c, http://imagej.nih.gov/ij).

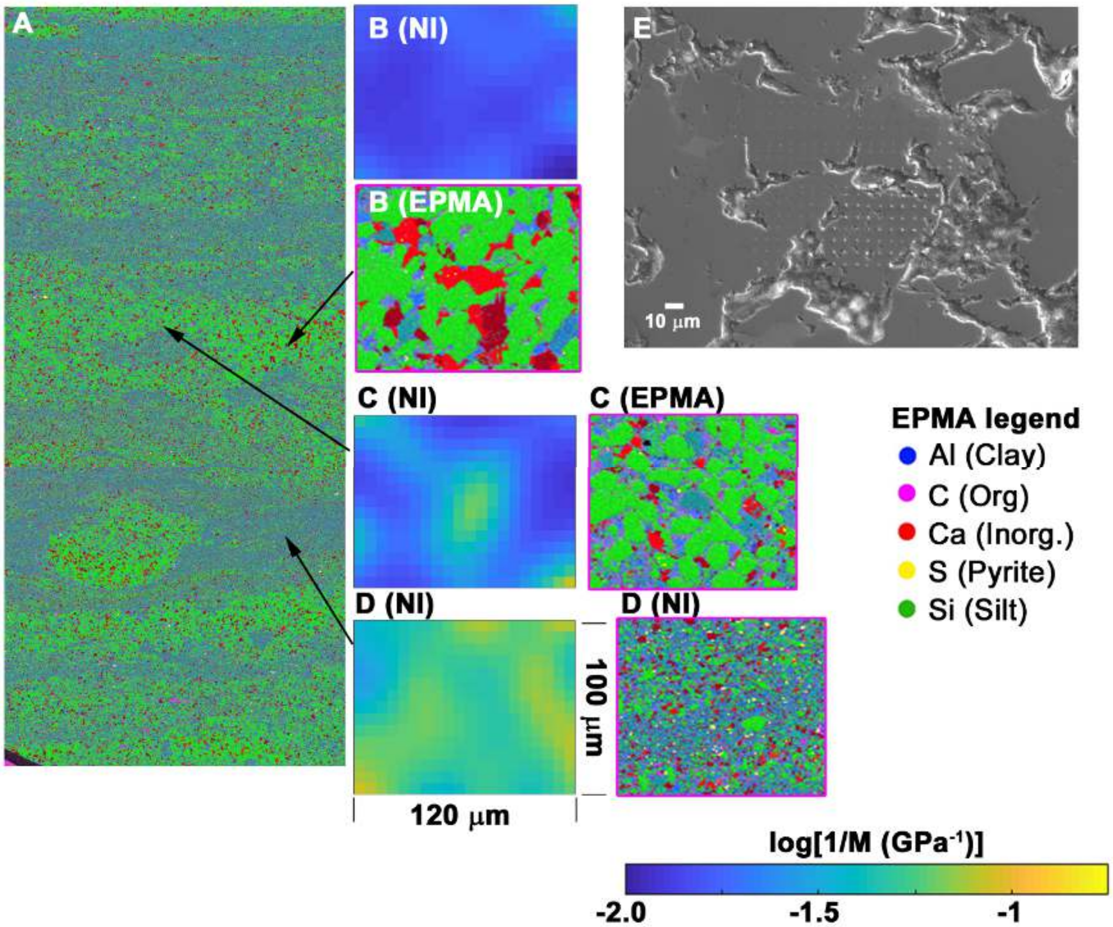

Fig. S- 6. Combined electron probe microanalysis (EPMA) and nanoindentation (NI) results for the Mancos OC sample. A) Areal EPMA map reproduced from **Fig. 1**. B.) Nanoindentation map and magnified view of EPMA map for small area (120 μm x 100 μm) characterized by high volume fraction of sand. C.) Nanoindentation map and magnified view of EPMA map for small area characterized by region where sand offers grain support to interstitial clay. D.) Nanoindentation map and magnified view of EPMA map for small area in argillaceous rock matrix. E.) Scanning electron microscope image of indentation map for area characterized by high volume fraction of sand (B). Comparing the size of the indentation divots to those shown in **Fig. S- 5**D indicates the compliance of the sand grains is much lower than that of the argillaceous matrix, especially in the vicinity of the microcrack **Fig. S- 5**B. The elemental map (A) is generated from the individual mass fractions using ImageJ (v. 1.53c, http://imagej.nih.gov/ij).

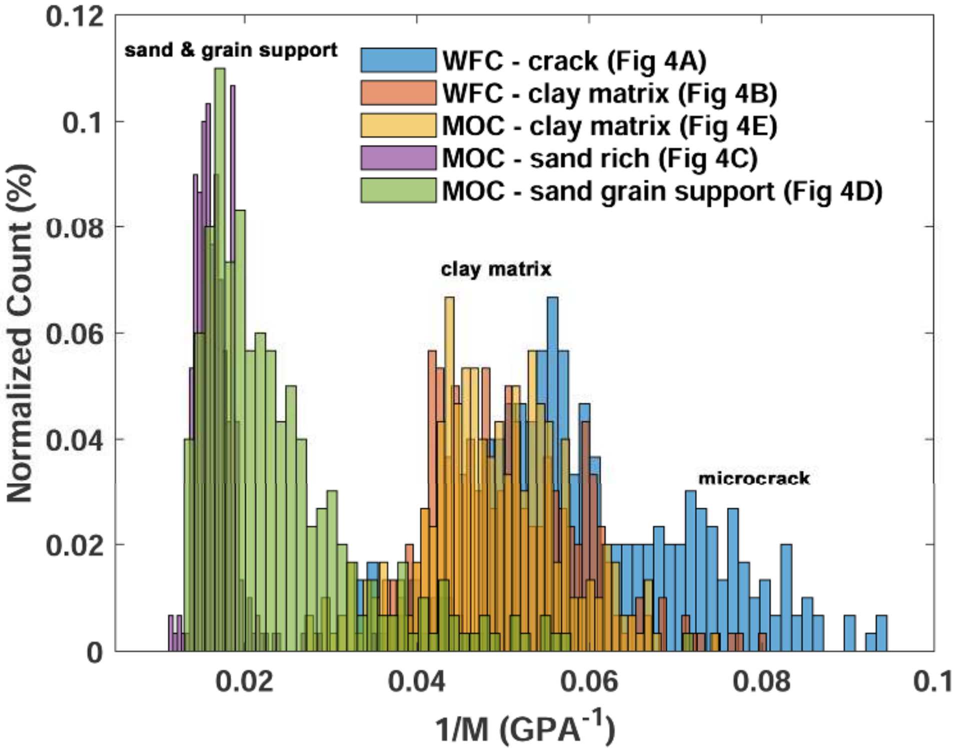

Fig. S- 7. Histogram of indentation compliance (1/M) for all maps shown in **Fig. 4**. The measurements were performed on the Wolfcamp (WFC) and Mancos OC (MOC) samples. The labels in the legend describe the microstructural constituent characterized by nanoindentation, and indicate the associated indentation maps as labeled in **Fig. 4**. The combined histogram plot clearly shows that the clay matrix component of the rock is characterized by a compliance that lies between that of the sand grains, the grain supported regions, and the region in the vicinity of the microcrack (WFC-crack).
